# A pilot prospective study to evaluate whether the bladder morphology in cystography and/or urodynamic may help predict the response to botulinum toxin a injection in neurogenic bladder refractory to anticholinergics

**DOI:** 10.1186/1471-2490-14-66

**Published:** 2014-08-14

**Authors:** Ronaldo Alvarenga Álvares, Ivana Duval Araújo, Marcelo Dias Sanches

**Affiliations:** 1 SARAH Network of Rehabilitation Hospitals, Unit Belo Horizonte, Minas Gerais, Av Amazonas 5953, Gameleira 30510-000, Brazil; 2 Federal University of Minas Gerais - UFMG, Rua Alfredo Balena, 190, 30130-100, Brazil

**Keywords:** Neurogenic detrusor overactivity, Botulinum toxin A, Cystography, Urodynamic, Neurogenic bladder

## Abstract

**Background:**

We have observed different clinical responses to botulinum toxin A (BTX-A) in patients who had similar urodynamic parameters before the procedure. Furthermore, some bladders evaluated by cystography and cystoscopy during the procedure had different characteristics that could influence the outcome of the treatment. The aim of this study was to assess whether cystography and urodynamic parameters could help predict which patients with neurogenic detrusor overactivity (NDO) refractory to anticholinergics respond better to treatment with injection of BTX-A.

**Methods:**

In total, 34 patients with spinal cord injury were prospectively evaluated. All patients emptied their bladder by clean intermittent catheterization (CIC) and had incontinence and NDO, despite using 40 mg or more of intravesical oxybutynin and undergoing detrusor injection of BTX-A (300 IU). Pretreatment evaluation included urodynamic, and cystography. Follow-up consisted of urodynamic and ambulatory visits four months after treatment. The cystography parameters used were bladder shape, capacity and presence of diverticula. Urodynamic parameters used for assessment were maximum cystometric capacity (MCC), maximum detrusor pressure (MDP), compliance and reflex volume (RV).

**Results:**

After injection of BTX-A, 70% of the patients had success, with 4 months or more of continence. Before the treatment, there were significant differences in most urodynamic parameters between those who responded successfully compared to those who did not. Patients who responded successfully had greater MCC (p = 0.019), higher RV (p = 0.041), and greater compliance (p = 0.043). There was no significant difference in the MDP (0.691). The cystography parameters were not significantly different between these groups bladder shape (p = 0.271), capacity (p > 0.720) and presence of diverticula (p > 0.999). Statistical analyses were performed using SPSS (version 20.0) and included Student’s t-test for two paired samples and Fisher’s exact test, with a significance threshold of 0.05.

**Conclusions:**

This study suggests that the cystography parameters evaluated cannot be used to help predict the response to injection of BTX-A in the treatment of refractory NDO. However, the urodynamic parameters were significantly different in patients who responded to the treatment, with the exception of the MDP.

## Background

Botulinum toxin (BTX), which was described by Van Ermengem [1] in 1897, exists as serotypes A, B, C, D, E, F and G [2]. Currently, serotypes A and B are available for clinical use. When injected into the muscle, BTX causes flaccid paralysis by inhibiting acetylcholine release at the presynaptic cholinergic junction. This effect is transient and dose-related. In smooth muscle, it was shown by Smith et al. [3] that BTX-A affects the release of acetylcholine and norepinephrine in the bladder and urethra, respectively.

The treatment of neurogenic detrusor overactivity (NDO) with an injection of BTX-A into the detrusor muscle was introduced in 2000 [4]. This therapy is a minimally invasive treatment option, and, although more invasive than oral treatment with anticholinergic, is less invasive than surgery [4]. Its safety and efficacy has been confirmed in a randomized, placebo-controlled clinical trial [5]. Some studies have evaluated the use of BTX-A injections in the detrusor muscle of patients with spinal cord injury to reduce NDO, increase bladder capacity, reduce incontinence and improve the quality of life of these patients [5,6].

In previous studies, we have observed different clinical responses to BTX-A in patients who had similar urodynamic parameters before the procedure [7]. This study was motivated by the observation that some bladders evaluated by cystography and cystoscopy during the procedure revealed different characteristics that could influence the outcome of the treatment. With respect to the morphology observed in cystography, we investigated whether differences in diverticula, shape, and capacity could influence the results of treatment. Similarly, we assessed whether the urodynamic parameters evaluated before the procedure could help predict the results of detrusor BTX-A injection for the treatment of NDO refractory to anticholinergics.

## Methods

**Figure 1:**
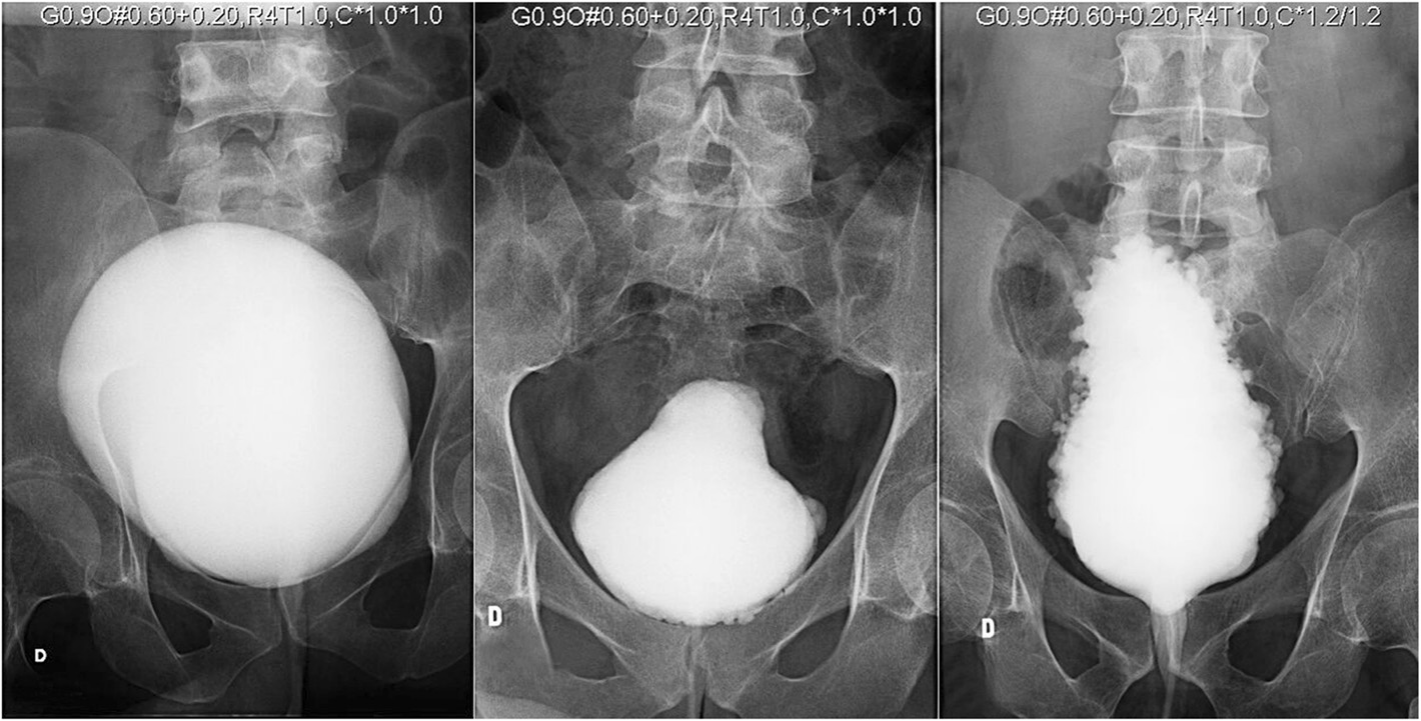
The shape of the bladder (“rounded” shape, “pear-shaped” or “pine”).

Thirty-four patients with spinal cord injury who received injections of BTX-A between January 2012 and July 2013 participated of this prospective observational study. The sample size was determined by G*Power software, version 3.1.7 [8] for two dependent groups (matched pairs). Parameters used in the calculations were as follows: effect size, 0.50; significance probability, 0.05; and power test, 0.80. Of the 34 patients, 23 were male and 11 were female. All methods and definitions were based on the standardization of terminology of lower urinary tract function, as described by Abrams et al. [9]. The study was approved by the ethics committee of Sarah Hospital in Brasilia (CAAE 24188413.3.0000.0022). Informed consent was obtained from all patients participating in the study.The inclusion criteria for the injection of BTX-A were those patients who emptied their bladder by CIC and had urinary incontinence due to hyperactivity refractory to intravesical oxybutynin doses equal or greater than 40 mg. An evaluation was conducted before the procedure and included a clinical history, physical examination, ultrasonography of the kidneys and urinary tract, cystography and urodynamic tests (Multichannel Urodynamics - Medtronic Duet systems, version 8.20, Minneapolis). The following urodynamic parameters were measured: reflex volume (RV), maximum detrusor pressure (MDP), bladder compliance, and maximum cystometric capacity (MCC). For better assessment of compliance, only the study bladders with a capacity ≥ 150 ml were used and compliance was measured five minutes after the end of bladder filling to allow for stabilization of the detrusor pressure. During cystography, the bladder filling was stopped when the maximum capacity had been reached, urinary leakage started, or there was supra pubic discomfort. Outcome was assessed in relation to the bladder shape and capacity and the presence of diverticula. The shape of the bladder was characterized as either “rounded” or “pear-shaped” and/or “pine” (see Figure 1). The cystometric capacity was measured in milliliters. Diverticula were characterized as absent or present, with one group consisting of patients with a diverticula number <10 and another group with a diverticula number ≥ 10 (Figure 1).

All procedures were performed in a hospital under general anesthesia. Antibiotics were administered orally, according to a urine culture, for seven days. The procedure performed on the fifth day of the antibiotic, all patients tested positive for bacteria. BTX- A (Westport Allergan Pharmaceuticals Ireland - Ireland) was diluted in sterile saline to a final concentration of 10 units/ml. Using a 19-Fr Storz cystoscope and 5 FR needle, a total of 300 IU (30 ml) was injected into 30 sites of the detrusor muscle, sparing the trigone region, as described in Schurch et al. [4]. Patients were instructed to continue using the anticholinergic medication and to gradually reduce the dose after the procedure, if it was successful and suspend its use if possible. The treatment was considered successful if the patient remained continent for four months or more, with complete absence of urinary leak, regardless of the use of the anticholinergic medication. Clinical and urodynamic control assessments were performed 4 months after treatment. Statistical analyses were performed using SPSS (version 20.0) and included Student’s t-test for two paired samples and Fisher’s exact test with a significance threshold of 0.05. Before each t-test, the hypothesis of equality of variances was checked using Levene’s test. Differences between the groups were compared with the Mann–Whitney U test for two independent samples.

## Results

Of the 34 patients, 23 were male and 11 were female. The mean age was 31.2 ± 10.3 years (mean ± standard deviation), with a range 19–55 years. There were 28 paraplegic and 6 tetraplegic patients and 25 traumatic and 9 non-traumatic cases. The mean time since SCI was 5.9 ± 4.4 years, with a maximum of 19 years and a minimum of 1 year. The patients tolerated all of the procedures and showed no acute complications related to the injections. Thirty patients (88.2%) were completely continent after the procedure. Six of these patients, however, showed little clinical response at four months and were considered unsuccessful. Four patients (11.8%) remained incontinent four months after treatment, although there were improvements in most urodynamic parameters and urinary losses. Twenty-four (70%) patients remained continent for more than 4 months and were considered successful. Twelve patients (35.2%) had an acontractile detrusor bladder after surgery. After four months, the following changes in the urodynamic parameters were observed: increased MCC (P <0.001), increased RV (p <0.001) and decreased MDP (p <0.001). The compliance did not change significantly (p = 0.366) (Table 1). There were significant differences in most of the pretreatment urodynamic parameters in patients with a successful response compared with those with an unsuccessful response. The MCC (p = 0.019), RV (0.041) and compliance (p = 0.043) were higher in patients with a successful response. The MDP was not significantly different between groups (p = 0.691) (Table 2).

**Table 1:** Urodynamic assessment before and after botulinum toxin injection

	**Before (n = 34)**	**After (n = 34)**	p-value^*^
Cystometric capacity (ml)	309 ± 155	492 ± 193	*<0.001*
Detrusor overactivity (cmH_2_O)	70 ± 27	41 ± 20	*< 0.001*
Reflex volume (ml)	228 ± 99	381 ± 229	*<0.001*
Compliance (ml/cmH_2_O)	32 ± 21	29 ± 18	=0.366

*Paired, two-sided Student’s t-test.

**Table 2:** Urodynamic parameters before botulinum toxin injection

**Urodynamic parameters**	**Success (n = 24)**	**Unsuccess (n = 10)**	p-value^*^
Cystometric capacity (ml)	335 ± 157	246 ± 138	0.019
Detrusor overactivity (cmH_2_O)	68 ± 27	72 ± 26	0.691
Reflex volume (ml)	243 ± 95	193 ± 103	0.041
Compliance (ml/cmH_2_O)	37 ± 23	20 ± 10	0.043

*Mann–Whitney U Test for two independent samples.

Regarding cystography, before the procedure, 24 patients (70%) had no diverticula, eight patients had between 1 and 10 diverticula and 2 patients had more than 10 diverticula. By Fisher’s exact test, there was no association between the response and the presence of diverticula (p > 0.999), bladder shape (p <0.271) or bladder capacity (p = 0.720) (Table 3).

**Table 3:** Results and cystography parameters

		**Success**	**Unsuccess**	p-value^*^
Capacity (ml)		351,0 ± 259,3 ml	315,8 ± 212 ml	P = 0,720
Forma	“Rounded” (n = 22)	17 (77%)	5 (23%)	P = 0,271
	“pear-shaped”/“pine” (n = 12)	7 (58%)	5 (42%)	
Diverticula	Absent (n = 24)	17 (71%)	7 (29%)	p > 0,999
	Present (n = 10)	7 (70%)	3 (30%)	

*Mann–Whitney U Test for two independent samples.

After 4 months of follow-up, twenty patients (58.8%) had reduced the dose of anticholinergics, five (14.7%) had discontinued their use and nine (26.5%) had not changed the dose (Table 4). Among the 24 patients with successful results, only 5 had discontinued the use of anticholinergics. However, 16 of these patients were able to decrease the dose of the anticholinergic drugs after the procedure (Table 5).

**Table 4:** Anticholinergic use after botulinum toxin injection (Total)

**Use of anticholinergics**	**N° patients**
Not decreased	9 (26,5%)
Decreased	20 (58,8%)
Suspended	5 (14,7%)
Total	34 (100%)

**Table 5:** Anticholinergic use after botulinum toxin injection (success)

**Use of anticholinergics**	**N° patients**
Not decreased	3 (12,5%)
Decreased	16 (66,6%)
Suspended	5 (20,8%)
Total	24 (100%)

## Discussion

BTX-A injections into the detrusor muscle provide a clinically significant improvement in patients with NDO refractory to anticholinergics and are very well tolerated (5;6). In this study, continence was observed over a period of more than 4 months in 70% of the patients undergoing treatment with BTX-A. In studies with similar populations of patients, the percentage of patients with continence after injection of the toxin ranged from 42 to 87% [10]. Karsenty et al. reported that anticholinergic agents may be discontinued in 28% to 58% of patients after treatment with BTX-A and the dose can be substantially reduced in the remaining patients [10]. In this study, after 4 months of follow-up, twenty patients (58.8%) had reduced the dose of anticholinergics, five (14.7%) discontinued the use and nine (26.5%) did not change the dose of medication (Table 4). Among the 24 patients with successful results, only 5 had discontinued the use of anticholinergics. However, 16 patients were able to decrease the dose of anticholinergic drugs after the procedure (Table 5). We note that some of these patients did not reduce the dose because they were afraid that the urinary losses would return after being continent. According to previous studies, some factors that may be related to the efficacy of BTX-A injections into the detrusor muscle include, for example, whether the doses of anticholinergics used before the procedure were considered high (refractory bladder) [7], what the optimal dose of BTX-A is [11,12], the formulations used [13,14] and the injection technique [11]. These factors may explain some of the differences observed in the results across different studies. The results obtained in this study were similar to earlier studies. Among patients presenting with NDO that is refractory to anticholinergic agents, there was a percentage with unsuccessful results.

Although it has been reported that alterations can occur in the detrusor muscle of the NDO [15,16], it was postulated that the morphology of the bladder (shape and presence of diverticula) could be a result of these alterations, and could, in turn, influence the response to BTX-A injections. These changes in bladder shape are most likely the result of smooth muscle hypertrophy and changes in the connective tissue matrix that do not respond to conservative treatment. However, the present study did not demonstrate that the cystography parameters could predict which cases would be more likely to have a better response to BTX-A injections.

Regarding urodynamic parameters, we observed in this study that the bladders that had better compliance, greater capacity and increased reflex volume before treatment showed a better response to treatment. Furthermore, there was no significant change in compliance after surgery (p = 0.366), suggesting that compliance is related to alterations in the bladder wall, is unresponsive to drug therapy, and is, therefore, directly related to the treatment response. This is in contrast to a study conducted by Klaphajone J [17], in which a small number of patients did not show this relationship.

## Conclusion

This study suggests that the cystography parameters evaluated cannot be used to predict the response to BTX-A injection for the treatment of refractory NDO. It was observed in the urodynamic parameters, that patients whose bladders had higher cystometric capacity, greater reflex volume and greater compliance showed better results after treatment. The bladder compliance showed no significant improvement after the procedure, which is an important factor to be noted. A larger study with a multivariate analysis would be appropriate to clarify the results of this work.

## Abbreviations

NDO: Neurogenic detrusor overactivity; BTX-A: Botulinum toxin A; BTX: Botulinum toxin; CIC: Clean intermittent catheterization; RV: Reflex volume; MDP: Maximum detrusor pressure; MCC: Maximum cystometric capacity.

## Competing interests

The authors declare that they have no competing interests.

## Author’s contributions

RAA conceived of the study and carried out the acquisition, analysis and interpretation of data and was involved in drafting the manuscript. IDA provided substantial contributions to the conception and design and revised the manuscript critically for important intellectual content. MDS participated in the study design and coordination. All authors read and approved the final manuscript.

## Pre-publication history

The pre-publication history for this paper can be accessed here:

http://www.biomedcentral.com/1471-2490/14/66/prepub
